# Forgiveness, dignity and hopelessness: the role of mediation and balance in MDD

**DOI:** 10.1192/j.eurpsy.2026.11428

**Published:** 2026-07-31

**Authors:** A. Franza, A. Litta, R. Longo, M. V. Minò, B. Solomita, A. Ricca, A. Vacca, F. Franza

**Affiliations:** 1 Neuroscientific Association “Neamente”, Naples - Avellino; 2 Cen.Stu.Psi., Provaglio d’Iseo (BS); 3 ”Città Solidale” Society - Cooperativa Sociale, Psychiatric Rehabilitation Center, Latiano (BR); 4 Psychiatric Rehabilitation Center “Villa dei Pini”, Avellino, Italy

## Abstract

**Introduction:**

Major depressive disorder is a very frequent illness that significantly compromises people’s well-being. Some factors, such as forgiveness, dignity, and hope, can be considered aspects that can reduce the course and severity of depressive illness. Forgiveness, both towards yourself and others, can improve emotional well-being and depressive symptoms. Dignity is an important element in maintaining self-esteem. Perceived loss of dignity or self-esteem can contribute to feelings of worthlessness and increase the risk of depression. Hopelessness is a powerful predictor of depression, which can lead to a pessimistic view of the future, worsening depression, and making recovery more difficult.

**Objectives:**

The aim of our observational “real world” study, which is part of a larger work, was to evaluate the role of forgiveness, dignity, and hopelessness in a group of patients suffering from MMD.

**Methods:**

83 patients were recruited [(females: 47; males: 36), mean age: total: 43.302 ±12.728; females: 42.720 ±13.698; males: 44.111 ±11.585)].

Inclusioni criteria: Total score HAM-D ≥14; PHQ-9 ≥5; all patients were in antidepressants treatment (paroxetine, clorimipramine, venlafaxine). All patients were administered at baseline (To), after 6 months (T1) and after 1 year (T2) the following rating scales:

- Patient Health Questionnaire-9 (PHQ-9)
- Heartland Forgiveness Scale (HFS) with subscales (Forginevess of Self (HFS-FS), Forgiveness of Others (HFS-FO), and Forgiveness of Situations (HFS-Si)
- Hamilton Rating Scale for Depression (HAM-D)
- Beck Hopelessness Scale (BHS)
- Patient Dignity Inventory (PDI)

Data were statistically analyzed with JASP and EZAnalyze 3.0

**Results:**

The data obtained showed a statistically significant reduction in depressive symptoms (HAM-D and PHQ-9 scales)(respectively, p<.001; p<.005). With HFS, a significant improvement in results was observed with the HFS-FS subscale (mean score T0: 19.19 vs T2:24.67, p:<.001), differences not statistically significant in the HFS-FO and HFS-Fsi subscales. The results obtained with the BHS showed a statistically significant difference T0 vs T2 (p: <.005). the results obtained with PDI also showed an improvement in the T0 vs T2 score.

**Conclusions:**

The complexity of depressive symptoms requires the importance of identifying some sentinel symptoms predictive of the disease. In our observational study, we identified and selected some aspects of forgiveness, dignity, and the absence of hope based on some studies previously carried out by our working group. The results of our study showed a correlation between improvements in self-forgiveness scores and reductions in depressive symptoms. However, no significant changes were observed in the other aspects of forgiveness. The data obtained on dignity and hope (or its absence) confirmed our previous studies. It is possible to hypothesize that psychotherapeutic work on these aspects can act positively on the symptoms of depression.

**Disclosure of Interest:**

None Declared

